# The *rarA* gene as part of an expanded RecFOR recombination pathway: Negative epistasis and synthetic lethality with *ruvB*, *recG*, *and recQ*

**DOI:** 10.1371/journal.pgen.1009972

**Published:** 2021-12-22

**Authors:** Kanika Jain, Elizabeth A. Wood, Michael M. Cox

**Affiliations:** Department of Biochemistry, University of Wisconsin-Madison, Madison, Wisconsin, United States of America; National Cancer Institute, UNITED STATES

## Abstract

The RarA protein, homologous to human WRNIP1 and yeast MgsA, is a AAA^+^ ATPase and one of the most highly conserved DNA repair proteins. With an apparent role in the repair of stalled or collapsed replication forks, the molecular function of this protein family remains obscure. Here, we demonstrate that RarA acts in late stages of recombinational DNA repair of post-replication gaps. A deletion of most of the *rarA* gene, when paired with a deletion of *ruvB* or *ruvC*, produces a growth defect, a strong synergistic increase in sensitivity to DNA damaging agents, cell elongation, and an increase in SOS induction. Except for SOS induction, these effects are all suppressed by inactivating *recF*, *recO*, or *recJ*, indicating that RarA, along with RuvB, acts downstream of RecA. SOS induction increases dramatically in a *rarA ruvB recF/O* triple mutant, suggesting the generation of large amounts of unrepaired ssDNA. The *rarA ruvB* defects are not suppressed (and in fact slightly increased) by *recB* inactivation, suggesting RarA acts primarily downstream of RecA in post-replication gaps rather than in double strand break repair. Inactivating *rarA*, *ruvB* and *recG* together is synthetically lethal, an outcome again suppressed by inactivation of *recF*, *recO*, or *recJ*. A *rarA ruvB recQ* triple deletion mutant is also inviable. Together, the results suggest the existence of multiple pathways, perhaps overlapping, for the resolution or reversal of recombination intermediates created by RecA protein in post-replication gaps within the broader RecF pathway. One of these paths involves RarA.

## Author summary

The RarA protein is part of a widespread protein family that is highly conserved from bacteria to humans. While the family clearly plays an important role in genome stability in all organisms, its molecular function remains undefined. Part of the reason for the lack of progress is genetic redundancy, where overlapping molecular functions render it difficult to discern the genetic effects of a gene when it is absent. In this study, we pinpoint a series of enzymes that must be absent to observe a strong effect of a deletion of the gene encoding RarA. In particular, a loss of RuvB function renders a *rarA* deletion highly sensitive to DNA damage. An additional loss of *recQ* or *recG* makes the cell inviable. Suppression of these defects by inactivation of proteins that load RecA protein indicate that RarA is somehow involved in the processing or reversal of branched DNA structures created by RecA and that RarA is part of an expanded version of the classic RecFOR pathway for repair.

## Introduction

DNA replication, indispensable to the survival and reproduction of all living organisms, is a highly coordinated and complex process. The progress of replication forks is regularly challenged by barriers extrinsic and intrinsic. These include DNA lesions produced by reactive oxygen species (ROS) or other DNA damaging agents and protein-DNA complexes. Encounters with such barriers can result in replication fork stalling or collapse. In some unknown fraction of encounters, a fork engages in “lesion-skipping”, disengaging and then re-initiating downstream and leaving the lesion behind in a post-replication gap [1–10]. These events represent a major source of mutagenesis and, if unresolved, can result in cell death. If post-replication gaps are not processed and closed prior to the next replication cycle, subsequent fork encounters will generate a double strand break.

Whereas the formation of post-replication gaps was among the earliest recognized outcomes of fork-lesion encounters [11–15], it remains arguably the most enigmatic. In principle, lesion-skipping prevents prolonged replisome stalling events. However, it is not known how often postreplication gaps are formed, how they are formed, how large they are, or what types of lesions are most proficient in triggering their formation. There appear to be three major paths for filling post-replication gaps in bacteria: (a) RecA-mediated homologous recombination [16–19], (b) translesion DNA synthesis [9, 17, 20], and (c) a RecA-independent template switching process [21–24]. Based on the effects of gene inactivation, RecA-mediated homologous recombination is probably the most important of these processes. However, RecA is multifunctional [25–28] and the proportion of the effect of a *recA* deletion that can be assigned to post-replication gap filling deficiency is difficult to parse.

In most cases, the primary pathway for the resolution of post-replication gaps is RecA-mediated recombinational repair via the RecFOR pathway [17]. Any pathway for recombinational DNA repair has three main steps. RecA is first loaded on a ssDNA substrate, normally pre-coated with SSB. In gaps, the loading of RecA onto SSB-coated ssDNA is facilitated by the RecO and RecR proteins, augmented by the RecF protein in a manner that has not yet been defined [25, 29–38]. The second step is pairing and exchange of homologous DNA strands by the loaded RecA nucleoprotein filament [39–42]. The joint molecules produced in this step are stabilized in some manner by the RecJ ssDNA 5′→3′ exonuclease [43–48]. The recombination intermediates created by RecA have the potential to interlink daughter chromosomes and block cell division. For cells to survive their creation, they must either be resolved or reversed in a third step. Resolution or reversal may be complex and context dependent. It is not clear that all the enzymes involved have been identified. The resolution/reversal phase is our focus in this report, along with proteins involved in it. Those proteins include the RuvABC proteins, the RecG and RecQ helicases, and the still enigmatic RarA protein.

In many cases, the RecA-mediated DNA strand exchange step can generate a four-armed Holliday junction. Processing of such junctions is a specialty of the RuvA, RuvB, and RuvC proteins, sometimes grouped in a complex called the RuvABC resolvasome [49–53]. RuvA is a Holliday structure-specific DNA binding protein. It interacts with and recruits the RuvB protein. RuvB is a powerful ATPase-driven DNA translocase. The RuvAB complex promotes a rapid and efficient branch migration [54–59]. The RuvC protein interacts with RuvAB [19, 60, 61]. RuvC is an endonuclease that interacts with RuvAB and introduces symmetrically opposed nicks across the branching point, resolving the Holliday junction into viable recombination products [49, 52, 62–64]. DNA ligation completes the process.

The RecG protein is a 76kDa SF2 multifunctional helicase. Although it can utilize a wider range of branched DNA substrates, RecG exhibits some functional overlap with the RuvAB branch migration complex. It carries a DNA-dependent ATPase activity that facilitates the remodeling of various branched DNA structures. In vitro studies of RecG have documented replication fork reversal [65–69] although a role in this process has not been confirmed in vivo [70, 71]. RecG is also involved in Holliday junction branch migration activity [72–76], resolution of structures formed in replication termination [77–79], and double strand break repair [80, 81]. Deletion of *recG* is linked with the increased accumulation of Holliday junctions at the replisome stalling site [82].

The existence of negative epistasis between the *ruvAB* and *recG* genes has been demonstrated [60, 72, 83, 84]. Cells devoid of either RuvAB or RecG alone display modest defects–small reductions in homologous recombination efficiency and weak sensitivity to DNA damage. However, a double deletion of the *ruv* genes and *recG* produces a synergistic decline in both parameters and reduces viability even in the absence of DNA damaging agents, suggesting that some redundancy exists in the function of the two systems [60, 72, 83, 84].

A second pathway for filling post-replication gaps is translesion DNA synthesis. This process is executed by specialized translesion DNA polymerases, including DNA polymerases II, IV, and V [17, 85]. DNA polymerase V is not normally present except when high levels of DNA damage led to an extended SOS response [86–89]. It is not clear how often DNA polymerases II and IV participate in the filling of post-replication gaps. Genetic results suggest that RecA-mediated recombination predominates the gap filling process [17].

The third pathway for post-replication gap filling is RecA-independent template switching. This process has been documented by examination of recombination events between relatively short, repeated DNA sequences [21, 22, 90, 91]. Although most of the homologous recombination in bacteria is carried out by RecA recombinase, a measurable level of recombination events has been documented in *ΔrecA* cells [21, 22, 90, 91]. This homology-dependent but RecA-independent recombination is increased in cells with defective DNA replication and restart pathways [24, 92].

The *Escherichia coli* RarA protein is required for most of this RecA-independent recombination [24]. RarA is a highly conserved AAA^+^ ATPase protein, sharing roughly 40% identity and 56–58% similarity with its *Saccharomyces cerevisiae* (Mgs1) and *Homo sapiens* (WRNIP1) homologs [93, 94]. The RarA protein family (RarA, Mgs1, and WRNIP1) is among the most widespread and conserved of any family with a putative role in DNA repair. Considerable research has been conducted on RarA and its homologs in the past two decades, but the molecular function of these proteins is still unknown. RarA family members are recruited to the replisome or nearby regions through an interaction either with SSB or PCNA [93–100]. They have been implicated broadly in genome maintenance [94, 101–109]. The RarA protein will bind to DNA gap or duplex ends and engages in an ATP-dependent DNA strand separation activity [110]. RarA is homologous to the DnaX clamp loader [93], but it functions as a tetramer [99]. These functional clues have not yet led to a demonstrable molecular function.

The RarA protein plays a prominent role in RecA-independent intermolecular recombination, especially at the regions carrying <200 bp homologous regions [24]. These events likely occur in post-replication gaps. However, the RarA-mediated recombination events are rare (much less frequent than RecA-mediated events), and it is not clear whether this is a major function of RarA or reflects an outcome that is incidental to the normal function of RarA.

The contribution of RarA to normal DNA metabolism has been largely overlooked due to the absence of major phenotypes accompanying its deletion from the cell. Inactivation of RarA has no significant impact on growth rate or sensitivity to DNA damaging agents [24]. However, the high degree of conservation evident in this gene family argues for a significant role. The answer may lie in genetic redundancy. RarA shares significant similarity not only with DnaX, the DNA polymerase III clamp loader, but also a 26% sequence similarity with RuvB. To explore the cellular role of RarA, we set out to define the genetic interactions of *rarA* with other known genes involved in DNA repair. In this screen, strong effects of a *rarA* deletion in a *ruvB* deleted background caught our attention and provided the genesis of the study presented here.

## Results

The following work examines the effects of *rarA* deletions and mutations in a variety of genetic backgrounds. We note that the *rarA* deletion strains described below utilize *rarA* ΔN406, which inactivates the gene by eliminating the first 406 codons but retains the final 40 codons of the gene. As will be described elsewhere, the final 40 codons of the gene include sequences that affect expression of the downstream gene *serS*. Deletion of the entire *rarA* gene affects *serS* expression and triggers a stringent response that can greatly obscure the normal effects of *rarA* gene inactivation. The protein has also been referred to as MgsA, a reference to its homology with the yeast protein Mgs1 [107]. As the RarA designation was proposed first [93], we use the *rarA* nomenclature.

### Elimination of both RarA and RuvB has a synergistic and deleterious effect on DNA metabolism

**Fig 1:**
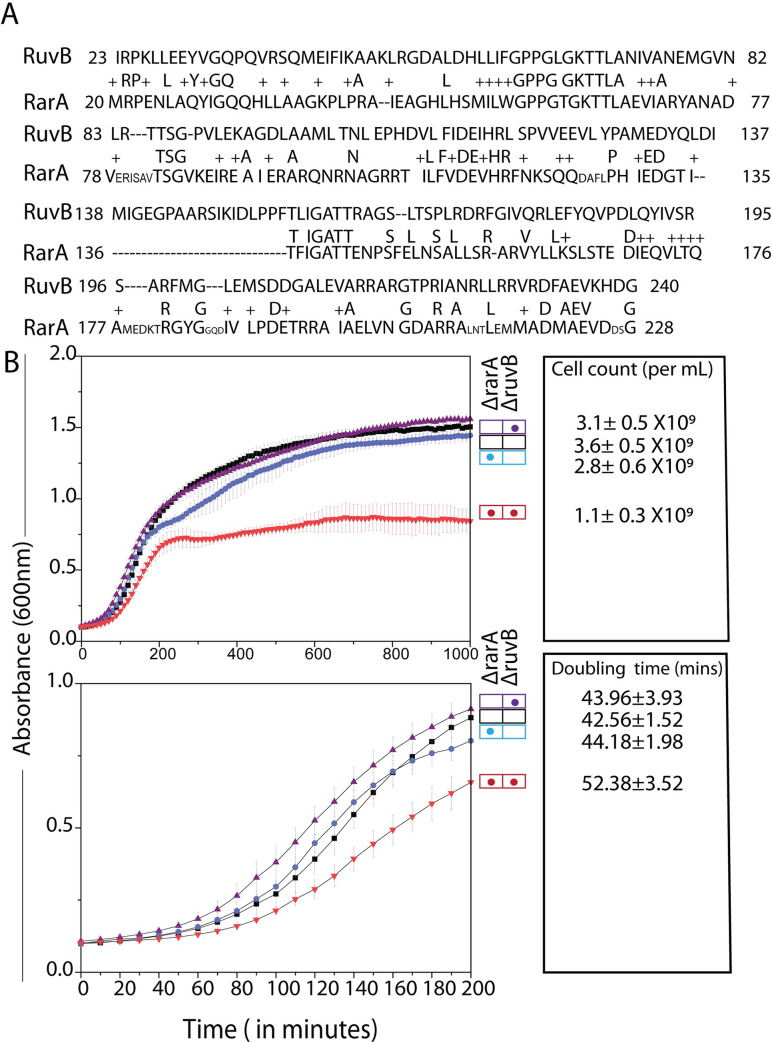
RarA shares similarity with RuvB protein and absence of both causes a growth defect. (A) RarA and RuvB share 26% sequence similarity as determined via BLAST alignment tool. (B) Elimination of RarA decreases the growth rate of *ΔruvB* cells. Growth curves for WT, *ruvB*, *rarA*, *ruvB rarA* grown at 37°C in LB medium for 1000 minutes are shown. Doubling time and cell counts per ml were calculated at exponential and stationary phase respectively. Error bars on the graph and in reported doubling times represent the standard deviation of at least three independent repeats carried out on the same day in the same microtiter plate. Each experiment was also repeated on three different days (each time in triplicate) with consistent results to confirm the phenotype.

The *Escherichia coli* RarA AAA+ ATPase protein shares 26% sequence identity and 46% similarity with the *E*. *coli* clamp loader DnaX [93]. RarA also shares 26% sequence identity and 44% similarity with the RuvB DNA translocase [93]. An alignment of *E*. *coli* RarA and RuvB via CLUSTAL X multiple sequence alignment illustrates the well-conserved nucleotide-binding sites with Walker A and Walker B motifs (Fig 1A). Our examination of the effects of *ruvB* deletions in a *rarA* deletion background revealed strong effects on many levels.

### Growth defect

We began by measuring the growth rate of Δ*rarA*, Δ*ruvB* and a double mutant Δ*rarA* Δ*ruvB* strain. The initial cultures were normalized to an OD_600_ of 0.005 and growth was monitored every 10 min for 24 h at 37°C. Deletion of *rarA* or *ruvB* alone does not significantly affect cell growth. However, deleting both *rarA and ruvB* causes significant growth defects (Fig 1B). The doubling time during exponential phase increased by approximately 10 min (Fig 1B). Stationary phase was reached much earlier, with only 1/3 the number of cells (measured by cfu) present.

### Sensitivity to DNA damaging agents

**Fig 2:**
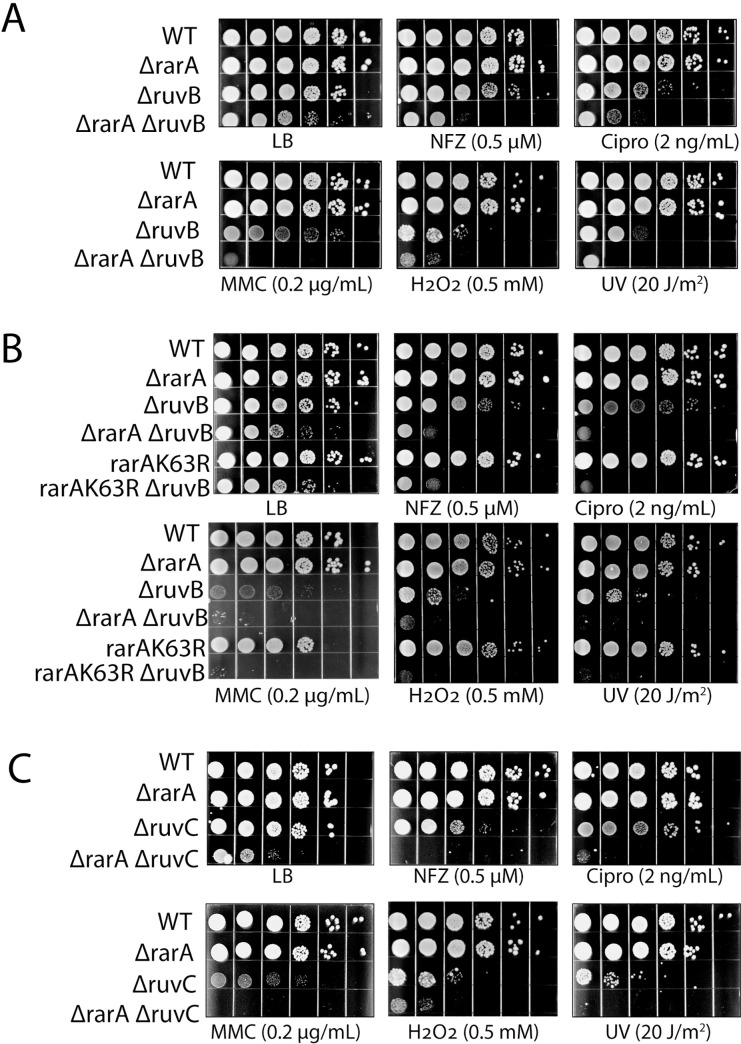
Synergistic sensitivity of strains lacking both RarA and RuvB/RuvC function to DNA damage. (A) Spot assays to test susceptibility of WT, *ruvB*, *rarA*, *ruvB rarA* (B) WT, *ruvB*, *rarA*, *ruvB rarA*, *rarAK63R*, *ruvB rarAK63R*, to test role of the RarA ATPase (C) WT, *ruvC*, *rarA*, *ruvC rarA* sensitivity. The concentration or dose of each DNA damaging agent is listed.

We tested the *ΔrarA*, *ΔruvB*, and *ΔrarA ΔruvB* strains for hypersensitivity to DNA damaging agents. Nitrofurazone (bulky guanine base adducts), ciprofloxacin (gyrase inhibitor), mitomycin C (DNA crosslinks), H_2_O_2_ (oxidation of Fe-S centers and Cys residues, plus strand breaks) and UV radiation (pyrimidine dimers), were all tested. Deleting *rarA* alone produced no significant defect on cell fitness with any of the DNA damaging agents at the concentrations employed (Fig 2A). Deleting *ruvB* alone produced modest effects on growth rate and/or viability with some of the DNA damaging agents. Elimination of both RarA and RuvB function increased the cells’ sensitivity to every DNA damaging treatment. A 2 to 3 log fold difference was evident in most cases between double and single mutants. Elimination of only the ATPase activity of RarA is sufficient to generate all of these effects (Fig 2B). Combining the *rarA* K63R mutant, which replaces a key residue in the Walker A box and eliminates ATP hydrolysis [99], with a *ruvB* deletion, replicates the results seen in the *ΔrarA ΔruvB* work almost exactly. The evident *rarA* epistasis seen with *ruvB* extends to *ruvC* (Fig 2C). The results suggest that, in concert with *rarA*, the response to DNA damage reflects the entire RuvABC system and not simply *ruvB*. The deleterious effects generally reflect decreased viability but slower growth rates also contribute, as seen when plates are incubated for longer times (S1 Fig)

These results indicate that RarA and RuvB exhibit some degree of functional homology that may reflect the existence of multiple pathways for resolution or reversal of recombination intermediates. Removal of both RarA and RuvB-dependent DNA repair processes greatly decreases the damage tolerance capacity of the cell and results in a growth defect in the absence of exogenous damage.

### Suppression of effects by elimination of *recF*, *recO* functions

**Fig 3:**
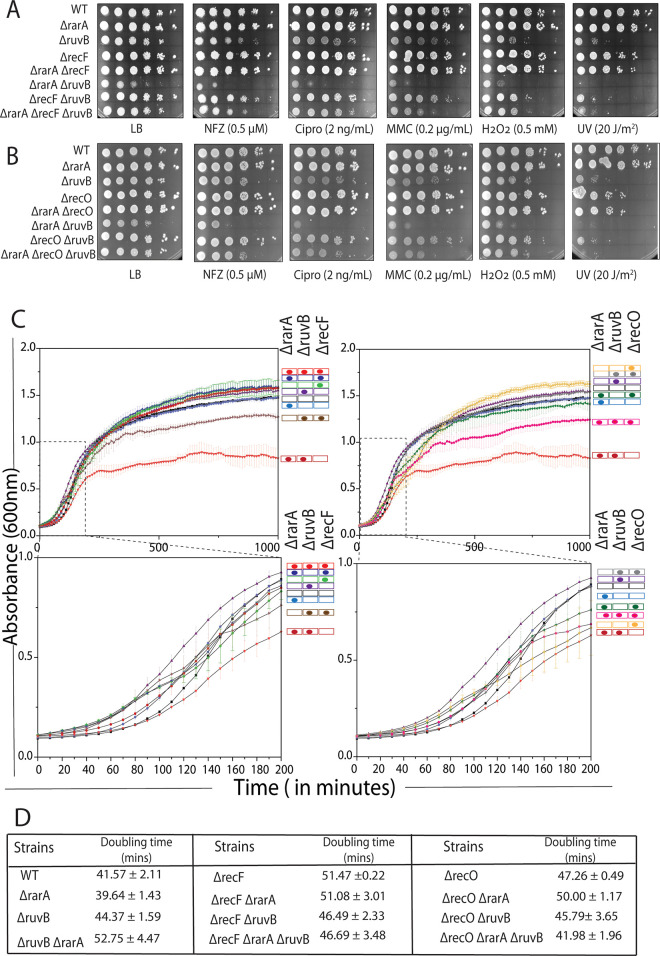
Suppression of *rarA ruvB* deficiencies by inactivation of RecF or RecO. (A and B) Sensitivity analysis of *ΔrecF* and *ΔrecO* deletion in *rarA*, *ruvB* and *rarA ruvB* backgrounds on exposure to different drugs. (C and D). Growth curves of WT, *recF*, *recF rarA*, *recF ruvB*, *recF rarA ruvB*, *recO*, *recO rarA*, *recO ruvB*, *recO rarA ruvB* mutants in LB medium. OD_600_ vs time for each strain is shown in comparison to a wild type (in black). Using the data from the exponential (log) phase of these growth curves, the doubling time of the different strains was calculated and is presented as a table. Error bars on the graph and reported doubling time represent the standard deviation of three independent repeats carried out on the same day in the same microtiter plate. Each experiment was also repeated on three different days (each time in triplicate) with consistent results to confirm the phenotype.

The function of the RuvABC complex is generally to process recombination intermediates created by RecA protein. A failure to process these intermediates has the potential for toxicity. As RecA protein loading is mediated by the RecF, RecO, and RecR proteins in single strand DNA gaps, we explored the effects of *recF* and *recO* deletions on the phenotypes observed for the *ΔrarA ΔruvB* double mutants. Results are presented in Fig 3.

In general, introduction of a deletion of *recF* or *recO* alone did not increase sensitivity to any DNA damaging agent at the levels employed, except for modest effects with UV irradiation observed previously [25, 37, 111–116]. We note that the absence of *recF* or *recO* function does result in sensitivity to higher levels of NFZ or MMC than used here [113, 117]. There was little to no effect of an introduction of *recF* or *recO* deletions on DNA damage sensitivity when introduced into strains carrying *rarA* or *ruvB* deletions alone. In contrast, large effects were seen when *recF* or *recO* were introduced to the *ΔrarA ΔruvB* double mutant to create triple mutants. In all cases, the *recF* and *recO* deletions strongly suppressed the effects of the double mutants, generally decreasing sensitivity to levels seen with *ruvB* deletions alone. For example, In Fig 3A and 3B, panel 3, when the cells were treated with Ciprofloxacin, the addition of *ΔrecF* or *ΔrecO* mutations suppressed the sensitivity of *ΔrarA ΔruvB* cells by 2 to 3 logs.

To confirm if the suppression of the *rarA ruvB* phenotype by *recF* or *recO* deletions reflects the function of RecA loading on ssDNA, we further tested the effect of reducing RecA concentration on the sensitivity of *ΔrarA ΔruvB* cells. A point mutation (T to C) in the first position of the six-nucleotide Pribnow box sequence of the *recA* promoter was introduced to reduce expression of the *recA* gene [118]. As observed with the *recF* and *recO* deletions, addition of this mutation rescued the damage sensitive phenotype of *rarA ruvB* cells (S2 Fig). The results suggest that both RarA and RuvB are acting downstream of RecA, and that toxicity is avoided if RecA-generated recombination intermediates are not formed. The suppression was also seen in the growth curves (Fig 3C and 3D). The growth defect was eliminated when a *recF* deletion was introduced, reduced when *recO* was deleted.

### Effects on cell filamentation

**Fig 4:**
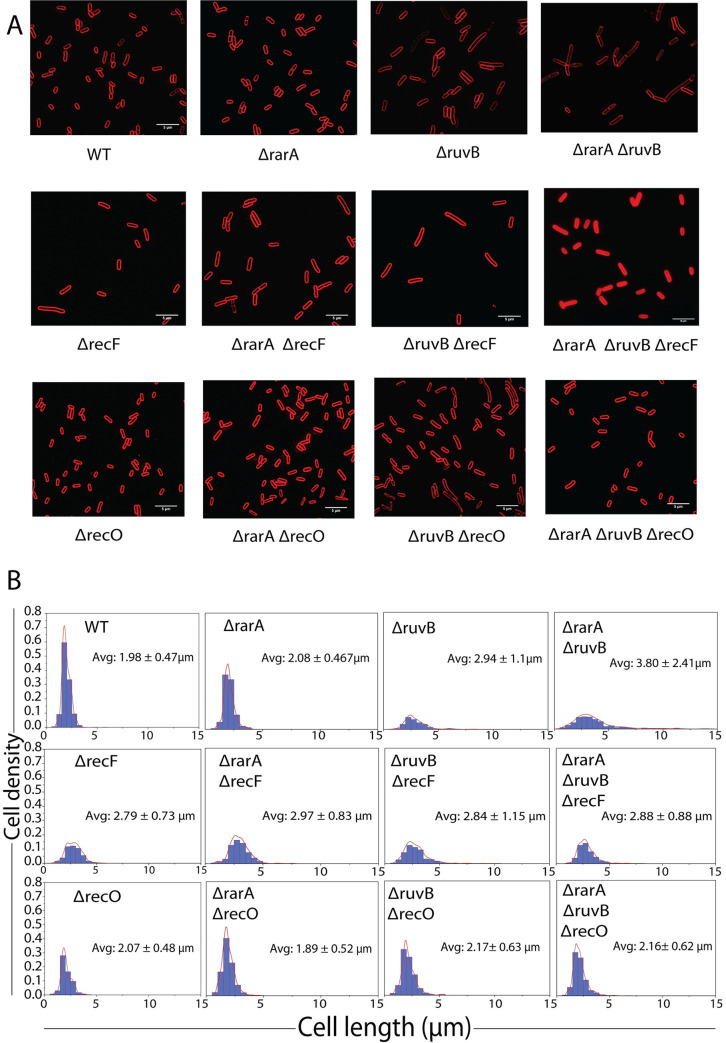
Deletion of RecF or RecO decreases the filamentation of *ΔrarA ΔruvB* cells. Deletion of *rarA* filaments the *ruvB-* cell. Morphological analysis of WT, *ruvB*, *rarA*, *ruvB rarA* was conducted using N-storm microscope as described in Methods. The effect of a deletion of *recF* or *recO* on the cell size of *rarA*, *ruvB* and *rarA ruvB* were also determined. Removal of *recF* or *recO* suppressed the filamentation defect of *rarA ruvB* cells. Reported error in cell length measurements represent the standard deviation of at least three independent repeats. n> 300 cells for each strain used.

The effects of *rarA* and *ruvB* function loss can also be seen in cell filamentation (Fig 4). Compared to wild type cells, loss of *rarA* or *recO* function has no discernible effect on cell length. Cells have a somewhat greater average length if *recF* or *ruvB* are inactivated. Loss of *rarA* and *ruvB* together has the largest effect on average cell length and results in the presence of a significant number of cells over 10 μm in length. The effects of the loss of *rarA* and *ruvB* were again suppressed by inactivating *recO* or *recF*. Loss of *recF* function in the double mutant decreases average cell length approximately to the level seen when *recF* alone was deleted. Loss of *recO* function in the double mutant decreases average cell length to the level seen in wild type cells.

### Suppression of effects by elimination of *recJ*

**Fig 5:**
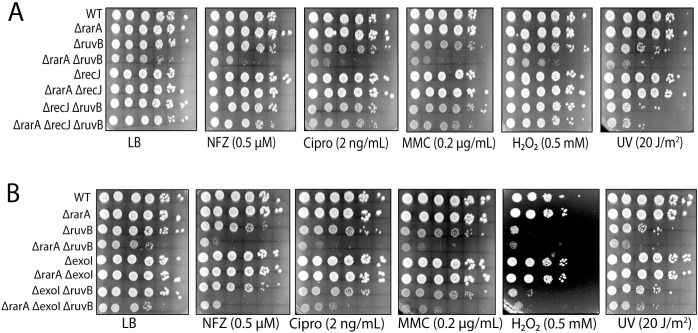
Deleting *recJ (5′-3′ exonuclease)* suppresses the sensitivity of *rarA ruvB* cells. (A) Susceptibility of *rarA*, *ruvB* and *rarA ruvB* with and without a *recJ* deletion to different drugs was tested (B) Deletion of Exonuclease I function has a small to negligible effect on the sensitivity of *rarA*, *ruvB* and *rarA ruvB* to damaging agents.

The RecJ protein is an exonuclease that degrades ssDNA 5′→3′, with a limited capacity to degrade into a duplex DNA [45, 119, 120]. RecJ has a role in stabilizing the formation of RecA-generated joint molecules [38, 43]. To complement our work with *recF* and *recO*, we examined the effects of deletions in the gene *recJ* along with another encoding the 3′→5′ ssDNA exonuclease *exoI*. The *recJ* deletion was essentially as effective as the *recF* and *recO* deletions in suppressing the sensitivity to DNA damaging agents (Fig 5A). The results suggest that RecJ plays an important role at early stages of RecA-mediated repair of post-replication gaps and that its action is toxic when the intermediates generated cannot be resolved or reversed by RarA or RuvB. Deleting the gene encoding exonuclease I had a modest suppression effect with respect to NFZ and ciprofloxacin (Fig 5B, panel 2 and 3), with a significant effect difficult to discern with MMC and peroxide. The effect of a *recJ* gene deletion is clearly greater.

### SOS induction

**Fig 6:**
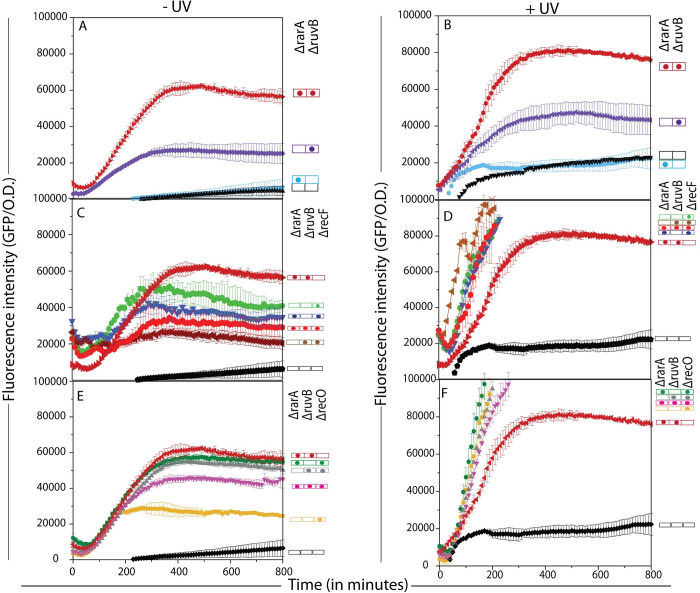
The SOS response is induced in *ΔrarA ΔruvB* cells, even in the absence of external stress. SOS induction profiling of different mutants with and without UV exposure was conducted. (A and B) SOS levels are increased substantially in *rarA ruvB* double mutants compared to any single mutants both with and without stress. SOS induction levels of (C and D) *rarA ruvB recF* and (E and F) *rarA ruvB recO* triple mutants were compared to their respective double and single mutants with and without UV exposure of 50J/m^2^. Addition of *ΔrecF* or *ΔrecO* partially suppresses the SOS levels of *rarA ruvB* cells under normal conditions. Error bars on the graph represent the standard deviation of three independent repeats carried out on the same day in the same microtiter plate. Each experiment was also repeated on three different days (each time in triplicate) with consistent results to confirm the phenotype.

We next explored whether the difference between the Δ*rarA* Δ*ruvB* and Δ*rarA* Δ*ruvB* Δ*recF* or Δ*recO* phenotypes to the different DNA damaging agents was also reflected in SOS induction levels. SOS induction requires the creation of ssDNA and the loading of RecA protein onto that ssDNA. Once loaded, RecA can both promote steps in recombination and also facilitate LexA cleavage to induce SOS [25, 27]. SOS induction is thus a (very) indirect indication of the presence of ssDNA as long as RecA is present and can be loaded. We used plasmid pEAW903 carrying SuperGlo GFP under the regulation of the SOS-inducible *recN* gene promoter. Deletion strains carrying pPrecN-gfp plasmid were grown to O.D. = 0.2. Replicate cultures were then treated or not with a UV dose of 50 J/m^2^. GFP expression along with absorbance was recorded every 10 mins for 16 h. SOS response was calculated by dividing the fluorescence intensity with OD for each time point to account for a difference in the growth rate in the strains tested. An absence of RarA function did not substantially alter the observed SOS signal, with or without UV treatment (Fig 6A and 6B). Elimination of RuvB function produced an increase in the SOS signal, both with or without UV treatment. Including a *rarA* deletion with the *ruvB* deletion resulted in a further increase in the SOS signals.

In the absence of UV, the *recF* or *recO* deletions produced substantial SOS signals on their own. Addition of deletions in *rarA* or *ruvB* somewhat decreased the signals seen with *recF*, whereas the signal seen with a *recO* deletion was increased by the presence of *rarA* or *ruvB* deletions. The *ΔruvBΔrarA* combination produced the greatest signal, and this was suppressed significantly by including either a *recO* or *recF* deletion. If the SOS signal is taken as a reflection of single-stranded DNA availability, a complex interplay is evident in which SOS may be reduced if RecA loading into gaps that occur during normal growth is limited and increased if RecA-generated intermediates in single-stranded DNA gaps cannot be processed properly.

When measured for the first hour after UV treatment, a loss of RecF or RecO function can result in slower SOS induction [37, 121]. This occurred in our system as well, although it was difficult to discern with SOS expression viewed over an extended period of time (Fig 6B, 6D and 6F). After that initial lag, deletion of *recF* or *recO* greatly increased the expression level of SOS-controlled genes even in the absence of UV or other induction. UV treatment of cells lacking *recO* or *recF* function can result in substantial genome degradation and very high SOS induction levels [122–125]. The presence of *rarA* and/or *ruvB* deletions did little to change the high levels of SOS induction (Fig 6B, 6D and 6F). The results suggest that RecA-mediated DNA repair, with loading from RecO and associated proteins, addresses the post-replication gaps created when UV lesion levels abruptly increase. If RecA loading into gaps is blocked by the absence of RecO or RecF, the SOS response is delayed and the gaps are not repaired. As a significant number of the cells survive (Fig 3), another repair path must eventually resolve some of the problems. This may be double strand break repair which is triggered by subsequent replisome encounters with the gaps leading to the generation of double strand breaks. Processing of these breaks by RecBCD and RecBCD-mediated loading onto the processed ssDNA could be responsible for the large increase in SOS induction seen after the first hour.

### Introducing a *recB* deletion to *ΔruvB ΔrarA* double mutant increases sensitivity to DNA damaging agents

**Fig 7:**
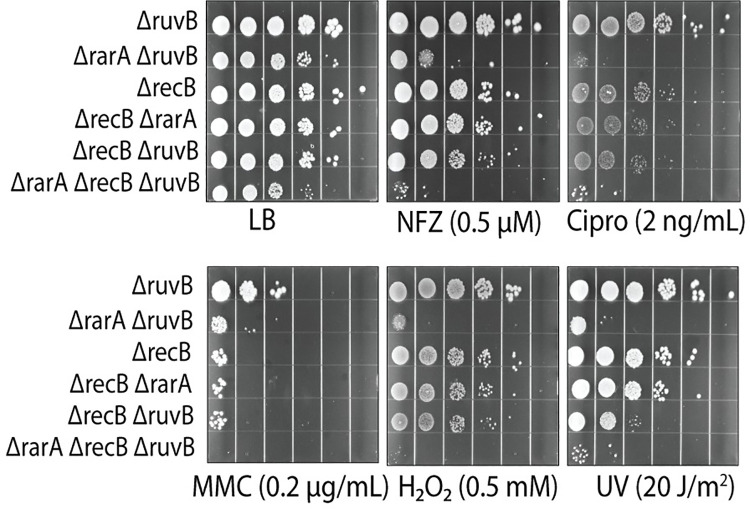
Addition of *recB* increases the sensitivity of *rarA ruvB* cells to DNA damage. Sensitivity check of *rarA*, *ruvB* and *rarA ruvB* cells with and without *recB* deletion towards various DNA damaging agents.

In double strand break repair, the enzyme that prepares the ssDNA and loads RecA protein is the RecBCD nuclease/helicase [126–128]. We wished to determine how the RecBCD repair pathway might be contributing to the observations described above for cells lacking *rarA* and *ruvB* function. Rather than suppressing the effects of a *ΔruvB ΔrarA* double mutant, the addition of a *recB* deletion to these cells increased sensitivity to most DNA damaging agents (Fig 7, row 4 and row 6). This suggests that (a) the toxic intermediates being produced by RecA protein and resolved by RarA and RuvB are not being produced during double strand break repair and (b) that most of the cells that survive high levels of DNA damage in a *ΔruvB ΔrarA* double mutant are relying on double strand break repair.

### Introducing a *recG* deletion to *ΔruvB ΔrarA* double mutant produces synthetic lethality

**Fig 8:**
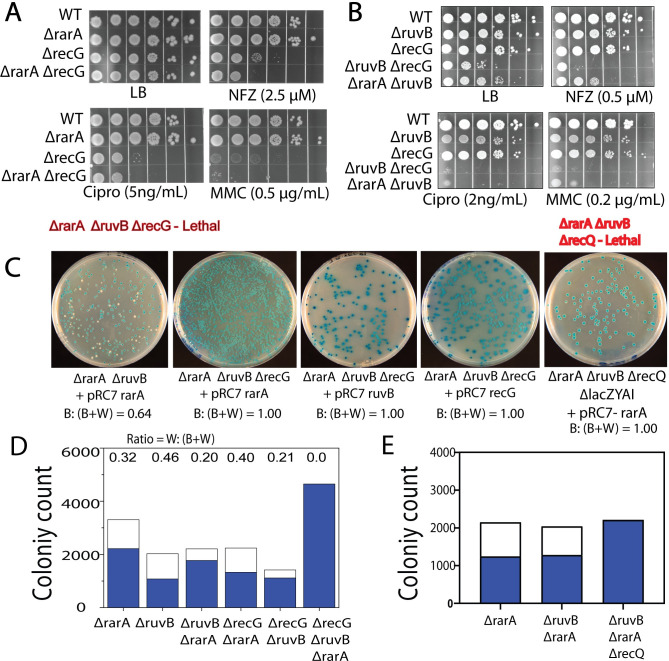
Deletion of *recG* or *recQ* renders *ΔruvBΔrarA* strains inviable. (A and B) Drug sensitivity analysis of a *recG* deletion in the absence of RarA or RuvB function. Deletion of *recG* in *ruvB* and somewhat *rarA* increases the cell sensitivity to many DNA damaging agents. (C) X-gal IPTG plate images showing the results of pRC7 assays in single, double and triple mutants of *rarA*, *ruvB*, *recG*, *and recQ* genes. pRC7 plasmid carrying the wild type copy of either *rarA*, *ruvB* or *recG* was employed. Many of the results are quantified in panels D and E. B: (B+W) = ratio of blue to total colonies.

We first checked if deletion of *rarA* alone affects the sensitivity of Δ*recG* cells substantially. Elimination of *rarA* modestly increases the sensitivity of *recG* cells to NFZ and MMC (Fig 8A). *ΔrecG ΔruvB* cells were extremely sensitive to all kinds of damage and exhibited reduced viability, as has been observed previously **(**Fig 8B**)** [60, 72, 83, 84].

We found it impossible to construct a triple mutant strain with deletions in the *rarA*, *ruvB* and *recG* genes. To confirm synthetic lethality, we used a mini-F plasmid-based assay to further characterize this genetic relationship. The unstable pRC7 plasmid that is employed in this study is a mini-F derivative that carries the lac operon and an antibiotic resistance gene for selection [118, 129, 130]. This unstable plasmid is rapidly lost in media without selection. In a *Δlac* background, cells containing the plasmid are blue when grown on X-gal and IPTG plates. Colonies arising from cells that lost the plasmid are white. Inserting a wild type allele of genes of interest on this plasmid will act as a form of selection in an antibiotic free media. We used three different pRC7 derivatives to validate our observation. Those were pJJ100, carrying a wild type copy of *recG*; pEAW1012, featuring a wild type copy of *rarA* and pEAW1193, a *ruvB*+ derivative of pRC7. Cells lacking either *rarA*, *ruvB* or *recG* alone or double mutants Δ*rarA* Δ*recG*, Δ*rarA* Δ*ruvB* or Δ*ruvB* Δ*recG* lost the plasmid expressing RarA and produced white colonies at a frequency range of 33%-50% after 24 hrs of growth (Fig 8C). To construct a triple mutant chromosomal background, we introduced the deletion only after transforming the cells with one of the pRC7 derivatives expressing one of the three proteins. When a *ΔrarA ΔrecG ΔruvB* strain was grown with pRC7 derivatives containing either *ruvB*, *rarA* or *recG* and plated on X-gal and IPTG plates, almost all of the colonies were blue after 24 h of growth, indicating strong plasmid retention. The results are quantified in Fig 8D. These observations indicate that RarA, RuvB and RecG share a functional relationship, and the presence of at least one is a prerequisite for cell survival.

### Introducing a *recQ* deletion to *ΔruvB ΔrarA* double mutant produces synthetic lethality

RecJ often acts in concert with the RecQ helicase to process DNA ends at double strand breaks [44, 46, 47, 82, 131, 132]. We thus determined what the effects of a *recQ* deletion would be in a *ΔrarA ΔruvB* background. Unlike the *recJ* deletions, the elimination of *recQ* function did not suppress the effects of a *ΔrarA ΔruvB* double mutant (S3A Fig). Instead, the *recQ* deletion, like the *recG* deletion, produced synthetic lethality (Fig 8C and 8E). In this genetic context, the effects of *recJ* and *recQ* are quite different as deletion of *recJ* in the *rarA ruvB* background did not increase the retention rate of pRC7-*rarA* plasmid as deletion of *recQ* clearly did (S3 Fig). Synthetic lethality with *ruvB* and *rarA* thus extends to the RecQ helicase, which has a function that is essential in a *ΔrarA ΔruvB* background to process intermediates produced by RecA, RecF, RecOR, and RecJ. RecQ can reverse RecA-mediated strand invasion [133] and this function may be especially important in *ΔrarA ΔruvB* cells.

### Elimination of RecF, RecO, or RecJ, suppresses the inviability of *ΔruvB ΔrarA ΔrecG* cells

**Fig 9:**
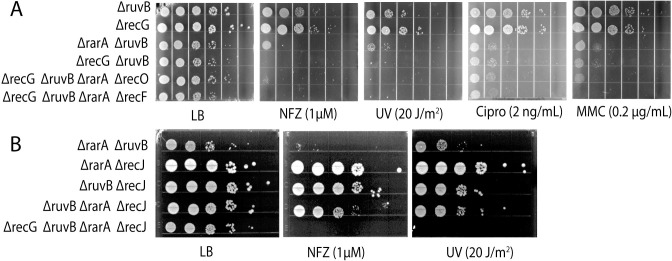
Addition of *recF*, *recO*, *or recJ* deletion restores the viability of the *rarA ruvB recG* mutant. (A) Effect of deletion of *recF* or *recO* on the viability and DNA damage sensitivity of *rarA ruvB recG*. (B) Effects of the deletion of *recJ* on the viability of the triple mutant.

Next, we determined if the loss of the RecA-loading system rescued viability in this triple mutant. The *recF* or *recO* deletions were first introduced into the *ΔruvB ΔrarA* background followed by the incorporation of a *ΔrecG* mutation. We observed that deletion of either *recF* or *recO* permits survival of *ΔruvB ΔrarA ΔrecG* triple mutant under normal growth conditions. However, these quadruple mutants displayed acute sensitivity to all DNA damaging agents. Thus, even though growth could be restored with this quadruple mutation under normal conditions in rich media, it remained highly sensitive to elevated levels of DNA damage (Fig 9A) as all of the main paths for DNA repair in gaps were lost. These results indicate that RecFOR mediated RecA-dependent recombinational DNA repair is toxic when RarA, RuvB, and RecG mediated resolution systems are all absent.

Deletion of *recJ* also suppressed the synthetic lethality of the *ΔruvB ΔrarA ΔrecG* triple mutant (Fig 9Β). This result helps to cement the role of RecJ in the creation of the intermediates that produce toxicity in cells lacking any pathway to resolve those intermediates.

## Discussion

**Fig 10:**
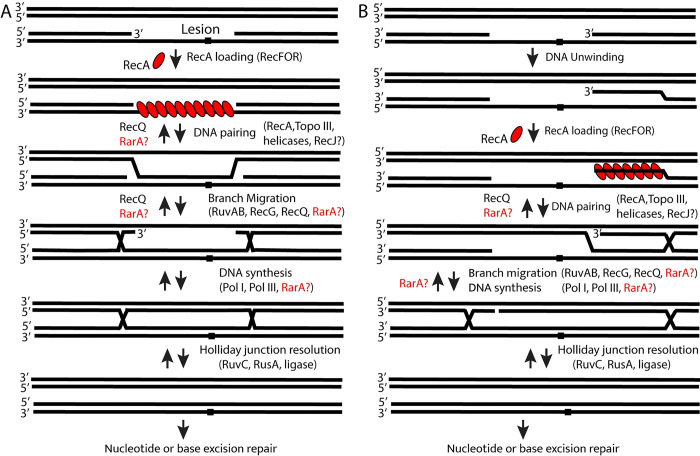
Recombinational DNA repair of a post-replication gap. This model is presented in part to illustrate the complexities inherent in using recombination to provide an undamaged strand against which a lesion in a post-replication gap can be repaired. (A) If RecA protein binds in the gap itself, as often presumed, DNA pairing and creation of a stable joint molecule must overcome a large topological barrier. (B) If RecA instead catalyzes strand invasion by an unwound 3′-ending DNA strand, topological barriers are still evident. A complex combination of enzymatic activities would be needed to support these reactions. There are many potential roles for undefined enzymatic activities such as RarA.

This work leads to two primary conclusions. First, loss of both RarA and RuvABC function causes a growth defect and synergistically increases cell susceptibility to almost all DNA damaging agents used in this study. This observation indicates that RarA exhibits some functional redundancy with the RuvABC proteins. The suppression observed by elimination of the RecF or RecO activities suggests a RarA function downstream of RecA protein. Second, the presence of both RecQ and RecG is essential in a cell lacking the function of RarA and RuvB. Deletion of either the *recG* or *recQ* genes in the Δ*rarA* Δ*ruvB* background makes the cell inviable. Again, if one assumes that RecF and RecO are needed to establish RecA filaments in post-replication gaps, suppression of the lethality by eliminating RecF or RecO suggests that in this genetic context, we are observing pathways that function in the resolution or reversal of intermediates created by the RecA recombinase. The role of RecJ in creation of those intermediates is less well understood but clearly important. Overall, the results suggest the presence of multiple pathways for resolution or elimination of RecA-generated intermediates during recombinational repair of post-replication gaps (Fig 10).

Our conclusion that the results of this study primarily reflect deficiencies in the repair of post-replication gaps is based on previous work with *rarA* [24] and the common assignment of the RecF and RecO proteins to gap repair [11, 34, 116, 134, 135]. All three of these proteins, RarA, RuvB, and RecG, have additional roles in DNA metabolism and some of the observed effects may reflect those roles in part. RarA is required for most RecA-independent recombination involving short DNA repeats [24]. This suggests an activity for RarA that may overlap with that of RecA [24, 107]. However, RecA-independent recombination is rare and RarA can support cell growth in a *ruvB recG* background. At first, the capacity of RarA to occasionally facilitate a RecA-like outcome when RecA is absent seems to conflict with a RarA function in the resolution or reversal of intermediates created by RecA. We suggest that the function of RarA downstream of RecA may be more important, with recombination in the absence of RecA merely incidental–a rare outcome of the still-enigmatic RarA activity. RuvB can function in double strand break repair as well as gap repair [58, 136–140] and it is not clear how much that role contributes to the deficiencies noted here. RecG has roles in post-replication gap repair [118, 141], but also in the regression of stalled forks [66, 68, 142–144] and avoiding over-replication at the replication terminus [78, 79, 145]. The suppression of effects here by elimination of RecF and RecO functions helps us focus on its role in post-replication gaps.

Decades of research has accorded the *recF*, *recO*, and *recR* genes the status of an epistasis group, often labeled RecFOR [31]. However, small differences observed in this work between the results seen with *recO* and *recF* should not be surprising. RecR forms complexes alternatively with RecO or RecF [16]. RecO, in complex with RecR, is clearly implicated in loading RecA protein onto SSB-coated ssDNA and promotes this reaction in vitro [16, 34–36]. Although RecF can affect (both positively and negatively) RecOR-mediated loading of RecA under limited conditions in vitro [16, 34–36], RecF or the RecFR complex does not facilitate RecA loading on its own. A complex containing all three proteins has never been observed. The RecF and RecO proteins rarely co-localize in vivo [31] and the molecular function of RecF is still enigmatic.

In principle, RecA-mediated gap filling can proceed in one of two ways (Fig 10). Both paths are replete with topological complexities. First (Fig 10A), the RecA filament may form on the ssDNA gap and promote strand invasion of that gap ssDNA into a homologous duplex. As the ssDNA bound by RecA in this instance has no free end, formation of a stable joint molecule would require the activity of a nuclease, topoisomerase, or both. Resolution of the stable joint molecule would entail additional topological problems as well as branch migration. Second (Fig 10B), the 3′-ending strand at one end of the gap can be used by RecA for strand invasion. This would require that RecA filaments NOT form in the gap but instead form on a 3′-ending strand created by a helicase and perhaps functions not yet defined. Stabilization and successful extension of the invading strand would again require the activities of a nuclease and/or a topoisomerase, as well as some function that would direct RecA to the unwound strand and away from the gap itself. Topoisomerase III has a role in post-replication gap repair via RecA-mediated recombination and could provide the topoisomerase function [114, 146–149]. In concert with RecQ, topoisomerase III can catenate DNA in a way that might facilitate one or both pathways in Fig 10 [147, 148]. Following strand invasion, if a break can be introduced into the displaced strand and the resulting 5′-ending strand degraded by RecJ, many of the joint molecule stabilization and topological problems inherent to these pathways could be alleviated. It is not clear what enzyme might contribute the postulated break or if it indeed occurs. There are many points in these pathways where RarA could participate, highlighted in Fig 10. They involve either the processing or reversal of branched DNA intermediates.

In the processing of double strand breaks, RecJ and RecQ can operate together to unwind the end (RecQ) and resect the 5′-ending strand [38, 47, 132]. In gap repair, the roles of these enzymes may be more distinct as suggested by these results, or not. RecQ may function with RecJ in the production or stabilization of joint molecules but its genetic effects as seen here could reflect a different function, the reversal of potentially toxic strand invasion events when their productive resolution is not possible. Alternatively, RecQ might collaborate with RecG in a recombination intermediate resolution process that requires both proteins.

RarA has sequence homology to both the RuvB DNA translocase and the DnaX clamp loader. The actual structure of the protein [99] suggests that the clamp-loader relationship could provide the more fruitful conceptual path. In both of the schemes of Fig 10, final repair of the gap will eventually require extension of a 3′ end by a DNA polymerase. In these pathways, there is no lesion in the template strand so polymerases I or III may be operative. A possible function for RarA would be clamp-loading to facilitate that step. Such a function has not been demonstrated to date, a failure that may reflect a requirement for a particular context of DNA structures and proteins not yet reconstituted. Alternatively, if strand invasion is followed closely by the installation of a DNA polymerase and a β-clamp to facilitate extension of the invading strand, then RarA might participate in reversal of that process by functioning as a clamp unloader. Of course, the function of RarA may be entirely different.

Overall, the results are reminiscent of the “death by recombination” observed by Rosenberg and coworkers in *ΔuvrD ΔrecG* or *ΔuvrD Δruv* strains [82]. Many of the same functions, including RecA, RecF, RecO, and RecR, are required to produce toxic recombination intermediates that are not resolved in these cells. RecQ and RecJ both contribute to the deleterious effects. In the *ΔrarA ΔruvB* background explored here, RecQ plays a supportive role that allows some cells to survive. We speculate that the capacity of RecQ to reverse RecA-mediated strand invasion may be more important in this background.

The results highlight once again the need for recombinational DNA repair during virtually every replication cycle when cells are grown in rich media, as noted elsewhere [7, 118]. Post-replication gaps also appear in virtually every replication cycle, even when added DNA damaging agents are not present. Resolution of these gaps is important and represents a first response to DNA lesions that the replisome has bypassed. Failure to initiate gap repair leads inevitably to double strand break repair when the subsequent replication fork arrives, with RecBCD providing a backstop to gap repair failures. Failure to complete gap repair can lead to fixation of DNA recombination intermediates behind the replication fork, which can block cell division and eventually trigger cell death.

With the links evident to RecF, RecO, and RecR, we propose that RarA should be added as a contributor to an expanded RecFOR recombination pathway for the repair of post-replication gaps. This expanded pathway should be viewed as one with multiple paths to the resolution of crossover intermediates created by RecA.

RarA, RuvB and RecG (or RecQ) provide parts of three pathways, perhaps overlapping, for the resolution or reversal of recombination intermediates created by RecAFORJ. RarA shares 56% homology with its human homolog, WRNIP1. In humans, WRNIP1 interacts with an ATP-dependent helicase gene called Werner helicase (WRN). WRN is a member of the RecQ DNA helicase family and is involved in many DNA transactions such as DNA recombination, repair, transcription, and telomere maintenance. Mutation in this gene causes the rare autosomal recessive disorder Werner’s syndrome, which is characterized by premature aging and early onset of age-related diseases [150–153]. The activity of WRNIP1 remains undefined, although it is clearly present in many key repair contexts [154–158]. We do not yet have a defined molecular function for RarA that conforms to its in vivo effects. A role in the processing or reversal of crossover intermediates created by RecA, as described above, seems likely based on the results of the current study. Ongoing research in bacteria should provide more detailed answers.

## Materials and methods

### Strain construction

All strains used in this study are *E*. *coli* MG1655 derivatives and are listed in Table 1. The *rarA*ΔN406 and *rarA*K63R strains were constructed using a galK+ selection-based recombineering method as described by Warming and Copeland [159]. The lamba red recombination method described by Datsenko and Wanner [160] was used to construct all other strains. When required, antibiotic resistance of a given strain was eliminated using FLP recombinase encoded by the pLH29 plasmid as described previously [161]. For strains containing multiple deletions, P1 transduction was used to introduce multiple alleles. To construct the quadruple mutant like *ΔrarA ΔruvB ΔrecF ΔrecG* strain, the *recF* deletion was first introduced into the Δ*rarA* Δ*ruvB* strain by P1 transduction. The *ΔrecG* mutation was then transduced into this strain via P1. All chromosomal mutations were confirmed by PCR amplification around all relevant deletion sites and/or using Sanger sequencing.

**Table 1:** List of strains used in this study.

Strain	Genotype	Parent strain	Source/Technique
**MG1655**	*rarA+ recA+ exoI+ recJ+ recF+ recO+ recR+ polB+ dinB+ umuDC+*		George Weinstock
**EAW1097**	Δ*ruvB*	MG1655	Transduction of MG1655 with P1 grown on **EAW401 (*ruvB*)**
**EAW1445**	Δ*ruvC*	MG1655	Lambda RED recombination
**EAW974**	Δ*rarA*	MG1655	**Gal K+ recombineering with no antibiotic markers**
**KJ642**	Δ*rarA ΔruvB*	EAW974	Transduction of Δ*rarA* with P1 grown on ***ΔruvB***
**KJ689**	Δ*rarA ΔruvC*	EAW974	Transduction of Δ*rarA* with P1 grown on ***ΔruvC***
**EAW907**	*rarAK63R*	MG1655	**Gal K+ recombineering with no antibiotic marker**
**KJ660**	*rarAK63R* Δ*r*uvB	EAW907	Transduction of *rarAK63R* with P1 grown on **Δ*ruvB***
**EAW629**	Δ*recF*	MG1655	Transduction of MG1655 with P1 grown on **Δ*recF***
**EAW114**	Δ*recO*	MG1655	Lambda RED recombination
**EAW989**	Δ*recF* Δ*rarA*	EAW974	Transduction of Δ*rarA* with P1 grown on **Δ*recF***
**EAW984**	Δ*recO* Δ*rarA*	EAW974	Transduction of Δ*rarA* with P1 grown on **Δ*recO***
**KJ746**	Δ*recF* Δ*ruvB*	EAW629	Transduction of Δ*recF* with P1 grown on **Δ*ruvB***
**KJ747**	Δ*recO* Δ*ruvB*	EAW114	Transduction of Δ*recO* with P1 grown on **Δ*ruvB***
**KJ691**	Δ*recF* Δ*rarA* Δ*ruvB*	KJ643	Transduction of Δ*ruvB* Δ*rarA* with P1 grown on **Δ*recF***
**KJ742**	Δ*recO* Δ*rarA* Δ*ruvB*	KJ643	Transduction of Δ*ruvB* Δ*rarA* with P1 grown on **Δ*recO***
**EAW820**	Δ*recJ*	MG1655	Lambda RED recombination
**EAW1150**	*ΔexoI*	MG1655	Transduction of Δ*rarA* with P1 grown on **EAW326 *(ΔexoI*)**
**EAW1147**	Δ*rarA* Δ*recJ*	EAW974	Transduction of Δ*rarA* with P1 grown on ***ΔrecJ***
**KJ773**	Δ*rarA* Δ*exoI*	EAW974	Transduction of Δ*rarA* with P1 grown on **Δ*exoI***
**KJ776**	Δ*ruvB* Δ*recJ*	EAW1097	Transduction of Δ*ruvB* with P1 grown on ***ΔrecJ***
**KJ772**	Δ*ruvB* Δ*exoI*	EAW1097	Transduction of Δ*ruvB* with P1 grown on **Δ*exoI***
**KJ775**	Δ*rarA* Δ*ruvB* Δ*recJ*	KJ643	Transduction of Δ*rarA* Δ*ruvB* with P1 grown on **Δ*recJ***
**KJ774**	Δ*rarA* Δ*ruvB* Δ*exoI*	KJ643	Transduction of Δ*rarA* Δ*ruvB* with P1 grown on **Δ*exoI***
**EAW102**	Δ*recB*	MG1655	Transduction of MG1655 with P1 grown on EAW81 (**Δ*recB)***
**EAW995**	Δ*recB* Δ*rarA*	EAW974	Transduction of Δ*rarA* with P1 grown on **Δ*recB***
**KJ696**	Δ*recB* Δ*ruvB*	EAW1097	Transduction of Δ*ruvB* with P1 grown on **Δ*recB***
**KJ694**	Δ*recB* Δ*rarA* Δ*ruvB*	KJ643	Transduction of Δ*ruvB* Δ*rarA* with P1 grown on **Δ*recB***
**EAW505**	Δ*recG*	MG1655	Lambda RED recombination
**KJ732**	Δ*recG* Δ*rarA*	EAW974	Transduction of Δ*rarA* with P1 grown on **Δ*recG***
**KJ666**	Δ*recG* Δ*ruvB*	EAW1097	Transduction of Δ*ruvB* with P1 grown on **Δ*recG***
**KJ883**	Δ*rarA* Δ*ruvB ΔlacZYAI +pRC7-rarA*	KJ879	Transformation of pRC7*-rarA* into Δ*ruvB* Δ*rarA ΔlacZYAI*
**KJ893**	Δ*recG* Δ*rarA* Δ*ruvB ΔlacZYAI +pRC7-rarA*	KJ887	Transduction of Δ*ruvB* Δ*rarA ΔlacZYAI* + pEAW1012 with P1 grown on **Δ*recG***
**KJ910**	Δ*recG* Δ*rarA* Δ*ruvB ΔlacZYAI +pRC7-ruvB*	KJ884	Transduction of Δ*ruvB* Δ*rarA ΔlacZYAI* + pEAW1193 with P1 grown on **Δ*recG***
**KJ894**	Δ*recG* Δ*rarA* Δ*ruvB ΔlacZYAI +pRC7-recG*	KJ879	Transduction of Δ*ruvB* Δ*rarA ΔlacZYAI* + pJJ100 with P1 grown on **Δ*recG***
**KJ923**	Δ*recQ* Δ*rarA* Δ*ruvB ΔlacZYAI +pRC7-rarA*	KJ887	Transduction of Δ*ruvB* Δ*rarA* + pEAW1012 with P1 grown on **Δ*recQ***
**KJ692**	Δ*recG* Δ*ruvB* Δ*rarA* Δ*recF*	KJ691	Transduction of Δ*ruvB* Δ*rarA* Δ*recF* with P1 grown on **Δ*recG***
**KJ736**	Δ*recG* Δ*ruvB* Δ*rarA* Δ*recO*	KJ735	Transduction of Δ*recG* Δ*rarA* Δ*recO* with P1 grown on **Δ*ruvB***
**KJ825**	Δ*recG* Δ*ruvB* Δ*rarA* Δ*recJ*	KJ802	Transduction of Δ*ruvB* Δ*rarA* Δ*recJ* with P1 grown on **Δ*recG***

### Growth curves and doubling time calculations

LB (3 ml) was inoculated with the indicated strains directly from the freezer stock to minimize the suppressor accumulation and growth before testing. Each culture was then diluted to give a starting OD_600_ of 0.005 and 100 μl of each culture was added to a 96-well plate. Growth was monitored at 37°C while shaking in a H1 Synergy Biotek plate reader. Optical density readings were taken every 10 min for 24 h. For doubling time calculations, semi-log curves of OD_600_ vs time were plotted. The slope (B) was estimated during the exponential phase for each strain. B is the slope of an exponential regression line for the semi-log curve. For example, if a quantity X increases from X_0_ at time t_0_ to 2*X_0_ at some future time t_0_ + Δt, Δt denotes the doubling time. Δt was calculated using the equation below,

$$\begin{array}{l} B=[\left(log(2)+log(X_{0}))‐log(X_{0}\right)]/\Delta t\\ B=log(2)/\Delta t. \end{array}$$

For cell count estimation, each strain was inoculated in LB to an OD_600_ of 0.01 and grown at 37°C till the OD_600_ reached 1.5 (stationary phase). A 1 mL culture aliquot of each strain was pelleted and resuspended in 1X PBS buffer. The cultures were serially diluted and 100μl of 10^−7^ and 10^−8^ dilutions of each culture were plated on LB plates. The total number of colonies was counted and total cell count per mL was estimated for each strain.

### SOS induction

To monitor SOS induction, a plasmid expressing SuperGlo GFP under the control of the early SOS *recN* promoter (pEAW903), was employed. Each strain was transformed with pEAW903 and the transformants were selected on ampicillin plates. Transformants were then inoculated in a 3 ml LB media containing ampicillin and the cultures were grown until they reached an OD_600_ of 0.2. Each culture was then split in two, and half of the culture of each strain was exposed to a UV dose of 50 J/m^2^. GFP fluorescence at 488/515nm and absorbance at 600 nm was then monitored every 10 mins for 24 h at 37°C using an H1 Synergy Biotek plate reader. SOS induction was calculated by dividing the GFP fluorescence values via absorbance. Statistical analysis was based on at least three replicates in all experiments.

### DNA damage sensitivity assay

All strains were grown in 3 ml LB culture overnight at 37°C with continuous shaking. 30 μl of overnight cultures of indicated strains were inoculated in fresh 3 ml LB medium and grown at 37°C until the OD_600_ measured 0.2. Aliquots (1 mL) were taken from each culture and were serially diluted in 1X PBS buffer (137 mM NaCl, 2.7 mM KCl, 10 mM Na_2_HPO_4_, 1.8 mM KH_2_PO_4_, 1 mM CaCl_2_ and 0.5 mM MgCl_2_) to 10^−6^. After each dilution, 10 μL were spotted on freshly made agar plates containing the indicated DNA damaging agents. Plates were incubated overnight at 37°C and imaged the next day using a 700 FOTO/Analyst Apprentice Digital Camera System (Fotodyne, Inc.). All experiments were repeated at least three times with comparable results.

### Mini-F pRC-7 plasmid assay

The pRC7 plasmid is a lac+ mini-F low copy derivative of pFZY1 [118, 129, 130]. Two derivatives of pRC7 were constructed, pEAW1012 that expresses a WT copy of the *rarA* gene and pEAW1193 that expresses a copy of *ruvB*. Another derivative, pJJ100 that harbors *recG*, was a generous gift from Christian Rudolph and was constructed as described previously [129, 130]. All indicated strains were transformed with pEAW1012, pEAW1193 or pJJ100 and selected on 0.5X ampicillin (Amp 50) plates before P1 transducing the final mutation in strains suspected of synthetic lethality. After P1 transduction, the cells were plated on Kan 40 and Amp50 plates to select for the cells that carry all the desired mutations with *rarA/recG/ruvB* copy expressed on a the appropriate pRC7 derivative. Following selection, overnight cultures of each strain were set in 3 ml LB media containing Amp 50 to select for the cells that retain the pRC7 plasmid. The next day, 5 ml fresh LB with no antibiotic was inoculated with 50ul of overnights. Cultures were grown till an OD_600_ reached 0.2. The culture was then placed on ice for 5 min followed by serial dilution in 1X PBS buffer and an appropriate dilution was spread on X-gal + IPTG plates. Plates were incubated at 37°C for 24 hrs, and the number of blue and white colonies were counted. All experiments were conducted at least three times and the total number of colonies counted was reported.

### Microscopy imaging

For all measurements of cell filamentation, a STORM/TIRF inverted microscope ECLIPSE Ti-E (Nikon), ORCA Flash 4.0 Hamamatsu camera and an oil objective (100X) was used. Image acquisition was performed at room temperature. Bright field and dsRed were used for image capturing. 0.16 mm thick borosilicate glass made coverslips (Azer scientific) were used for these experiments. Cells were grown overnight at 37°C in LB media. Secondary cultures were then reset using overnight saturated culture, 30 μl of overnight in 3ml LB media, and grown out till OD reaches 1.0. The cultures were then pelleted down, and cells were suspended in 1XPBS buffer. 2 μl of FM-64 dye (conc. 0.33M) were then added in 200 μL of culture and incubated on ice for at least 30 mins. For imaging, 2 μl of this mixture was loaded on the coverslip and covered with an agar pad (1.5% agarose in dH_2_O). A single bright field image (100ms exposure) and dsRed image (50 ms exposure) was taken at multiple fields of view. All experiments were repeated in triplicates and at least 300 cells were counted and analyzed for each strain.

## Supporting information

S1 Fig Distinguishing between the slow growth and reduced viability phenotype of different strains when treated with different DNA damaging agents. (A and C) Sensitivity analysis of *rarA*, *ruvB*, *ruvC*, *rarA ruvB*, *andrarA ruvC* cells towards various DNA damaging agents. Deletion of *rarA* in *ΔruvB* or *ΔruvC* cells slows growth at relatively low concentrations of DNA damaging agents and affects viability at higher concentrations. (B and C) Deletion of *recF*, *recJ*, *or recO* in *rarA ruvB* cells rescues its cell growth and viability under different DNA damaging conditions. Deletion of *recB* in *rarA ruvB* cells reduces viability.
(TIF)

S2 Fig Addition of *precA* decreases the sensitivity of *rarA ruvB* cells to DNA damage. Sensitivity analysis of *rarA*, *ruvB*, and *rarA ruvB* cells with decreased levels of *recA* towards various DNA damaging agents.
(TIF)

S3 Fig Difference between *recQ* and *recJ* deletions on *rarA*, *ruvB*, *and rarA ruvB* phenotype. (A) Sensitivity analysis of *rarA and ruvB* cells with *recQ* deletion towards various DNA damaging agents. Deletion of *recQ* does not affect the sensitivity of *rarA* or *ruvB* cells to different damaging agents, like *recJ*. (B) Deletion of *recJ* in *rarA ruvB* cells does not decreases the retention rate of pRC7-*rarA* plasmid, like *recQ*.
(TIF)

S1 Data This document provides the raw data for the experiments shown in Figs 1, 3, 4, 6 and 8. (XLSX)
