# Supplementary figures and images for: The rarA gene as part of an expanded RecFOR recombination pathway: Negative epistasis and synthetic lethality with ruvB, recG, and recQ
Source: PLoS Genet. 2021 Dec 22;17(12):e1009972. doi: 10.1371/journal.pgen.1009972 (PMC8735627; doi:10.1371/journal.pgen.1009972)

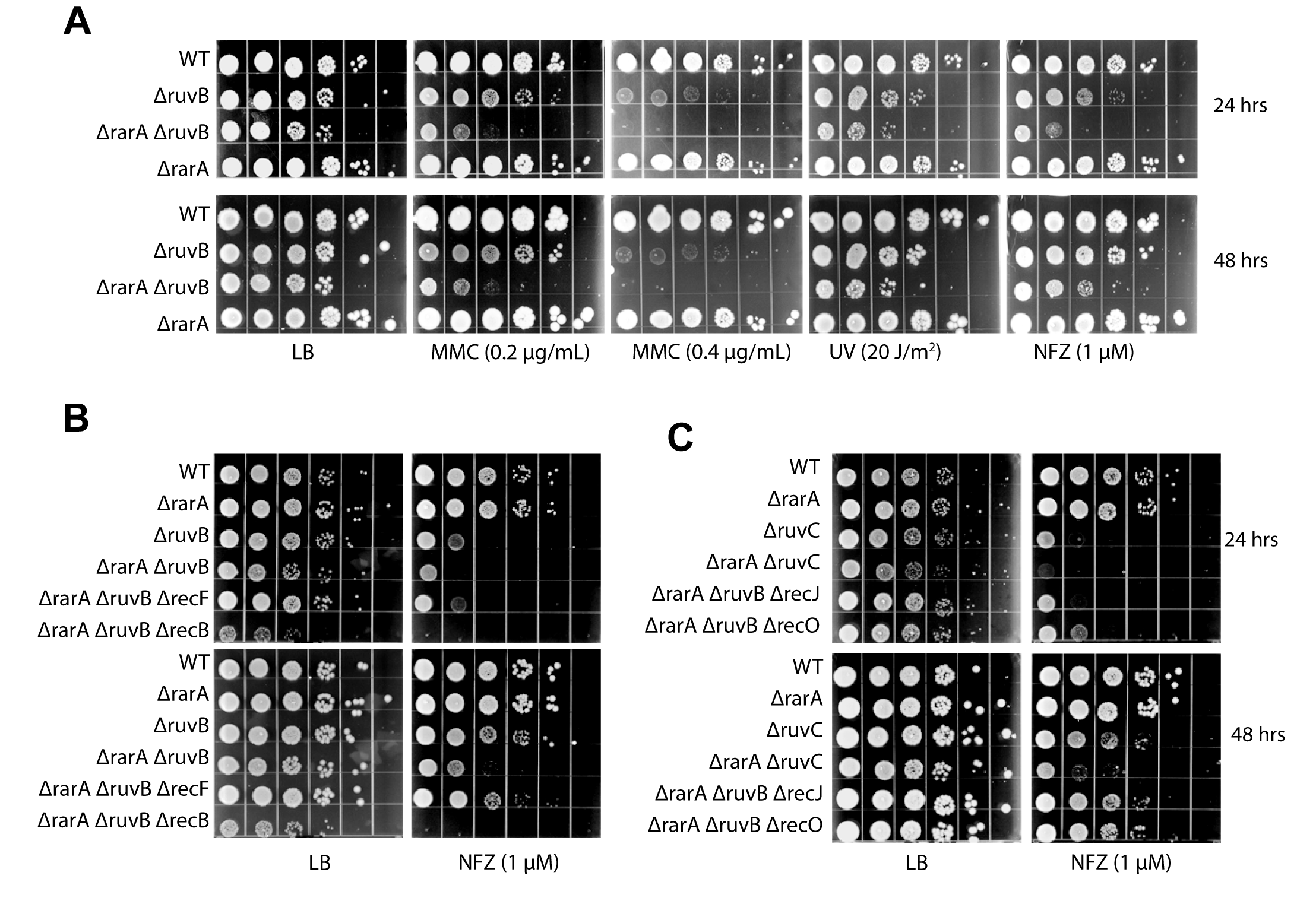

Supplement: S1 Fig — (A and C) Sensitivity analysis of rarA, ruvB, ruvC, rarA ruvB, andrarA ruvC cells towards various DNA damaging agents. Deletion of rarA in ΔruvB or ΔruvC cells slows growth at relatively low concentrations of DNA damaging agents and affects viability at higher concentrations. (B and C) Deletion of recF, recJ, or recO in rarA ruvB cells rescues its cell growth and viability under different DNA damaging conditions. Deletion of recB in rarA ruvB cells reduces viability. (TIF) [file pgen.1009972.s001.tif]

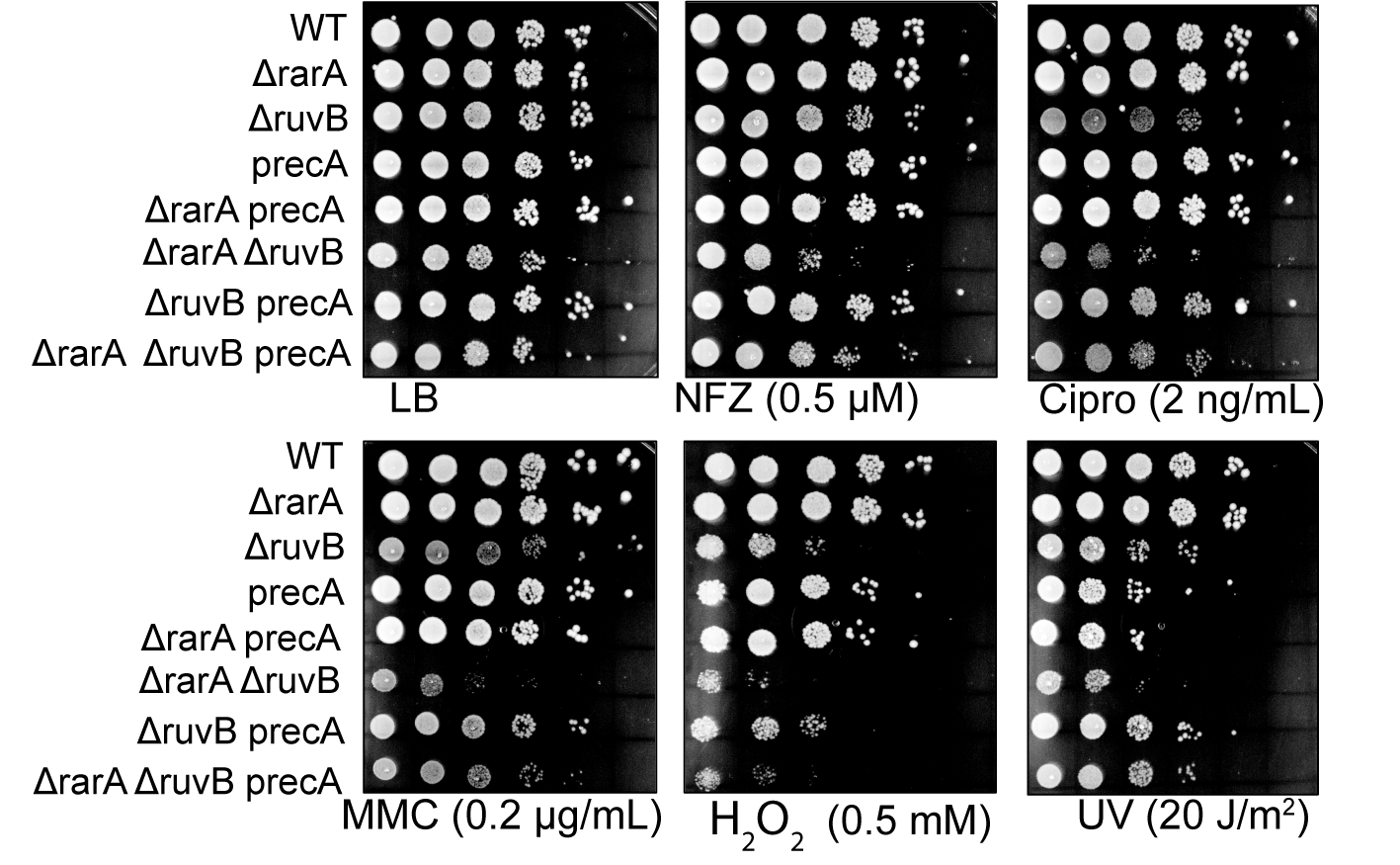

Supplement: S2 Fig — Sensitivity analysis of rarA, ruvB, and rarA ruvB cells with decreased levels of recA towards various DNA damaging agents. (TIF) [file pgen.1009972.s002.tif]

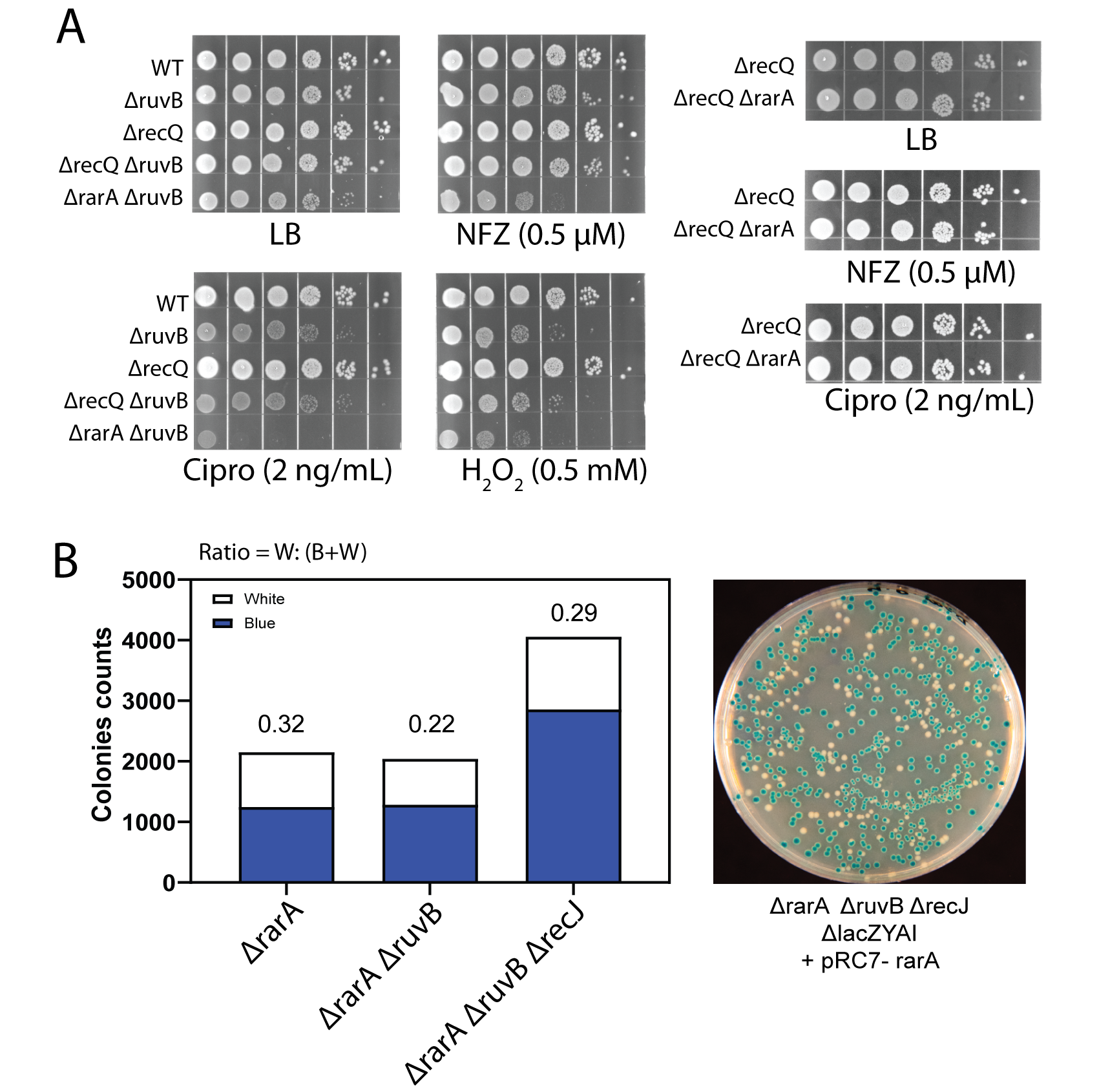

Supplement: S3 Fig — (A) Sensitivity analysis of rarA and ruvB cells with recQ deletion towards various DNA damaging agents. Deletion of recQ does not affect the sensitivity of rarA or ruvB cells to different damaging agents, like recJ. (B) Deletion of recJ in rarA ruvB cells does not decreases the retention rate of pRC7-rarA plasmid, like recQ. (TIF) [file pgen.1009972.s003.tif]
